# Factors Associated With the Availability of Medications for Opioid Use Disorder in US Jails

**DOI:** 10.1001/jamanetworkopen.2024.34704

**Published:** 2024-09-24

**Authors:** Elizabeth Flanagan Balawajder, Lori Ducharme, Bruce G. Taylor, Phoebe A. Lamuda, Marynia Kolak, Peter D. Friedmann, Harold A. Pollack, John A. Schneider

**Affiliations:** 1 Public Health Department, NORC at the University of Chicago, Chicago, Illinois; 2 National Institute on Drug Abuse, National Institutes of Health, Bethesda, Maryland; 3 Department of Geography and Geographic Information Science, University of Illinois at Urbana-Champaign; 4 Office of Research, Baystate Health and University of Massachusetts Chan Medical School-Baystate, Springfield, Massachusetts; 5 Crown Family School of Social Work, Policy and Practice, University of Chicago, Chicago, Illinois; 6 Department of Public Health Sciences, University of Chicago, Chicago, Illinois; 7 Department of Medicine, University of Chicago, Chicago, Illinois

## Abstract

**Importance:**

In 2023, more than 80 000 individuals died from an overdose involving opioids. With almost two-thirds of the US jail population experiencing a substance use disorder, jails present a key opportunity for providing lifesaving treatments, such as medications for opioid use disorder (MOUD).

**Objectives:**

To examine the prevalence of MOUD in US jails and the association of jail- and county-level factors with MOUD prevalence using a national sample.

**Design, Setting, and Participants:**

This survey study used a nationally representative cross-sectional survey querying 1028 jails from June 2022 to April 2023 on their provision of substance use disorder treatment services. The survey was conducted via mail, phone, and the internet. County-level data were linked to survey data, and binary logistic regressions were conducted to assess the probability that a jail offered any treatment and MOUD. A stratified random sample of 2791 jails identified by federal lists of all jails in the US was invited to participate. Staff members knowledgeable about substance use disorder services available in the jail completed the survey.

**Exposures:**

US Census region, urbanicity, jail size, jail health care model (direct employees or contracted), county opioid overdose rate, county social vulnerability (measured using the Centers for Disease Control and Prevention 2020 Social Vulnerability Index summary ranking, which ranks counties based on 16 social factors), and access to treatment in the county were assessed.

**Main Outcomes and Measures:**

Availability of any type of substance use disorder treatment (eg, self-help meetings), availability of MOUD (ie, buprenorphine, methadone, and naltrexone) to at least some individuals, and availability of MOUD to any individual with an OUD were assessed.

**Results:**

Of 2791 invited jails, 1028 jails participated (36.8% response rate). After merging the sample with county data, 927 jails were included in analysis, representative of 3157 jails nationally after weighting; most were from nonmetropolitan counties (‭1756 jails [55.6%; 95% CI, 52.3%-59.0%]) and had contracted health care services (1886 jails [59.7%; 95% CI, 56.5%-63.0%]); fewer than half of these jails (1383 jails [43.8%; 95% CI, 40.5%-47.1%]) offered MOUD to at least some individuals, and 405 jails (12.8%; 95% CI, 10.7% to 14.9%) offered MOUD to anyone with an OUD. Jails located in counties with lower social vulnerability (adjusted odds ratio per 1-percentile increase = 0.28; 95% CI, 0.19-0.40) and shorter mean distances to the nearest facility providing MOUD (adjusted odds ratio per 1-SD increase, 0.80; 95% CI, 0.72-0.88) were more likely to offer MOUD.

**Conclusions and relevance:**

In this study, few jails indicated offering frontline treatments despite being well positioned to reach individuals with an OUD. These findings suggest that efforts and policies to increase MOUD availability in jails and the surrounding community may be associated with helping more individuals receive treatment.

## Key Points

**Question:**

To what extent and to whom do US jails provide evidence-based treatment for opioid use disorder in their facilities?

**Findings:**

In this survey study of 1028 jails, less than half of jails (43.8%) offered medications for opioid use disorder to at least some individuals and 12.8% offered these medications to anyone with an opioid use disorder who requested them.

**Meaning:**

These findings suggest that many individuals with an opioid use disorder are not receiving necessary treatment while in jail.

## Teaser

This survey study investigates the prevalence of medications for opioid use disorder in US jails and factors associated with their availability.

## Introduction

With more than 80 000 fatal overdoses involving opioids in 2023,^1^ the US opioid crisis calls for the implementation of evidence-based public health interventions and policies.^2,3^ Correctional settings present key opportunities for such interventions. Opioid use is associated with criminal-legal system involvement.^4^ Nearly two-thirds of the US jail population is estimated to experience an active substance use disorder (SUD).^5^ A county-level study^6^ found that 21% of individuals who died of a fatal overdose had recently been in the county jail. Medications for the treatment of opioid use disorder (MOUD) is an evidence-based approach that involves treatment with 1 of 3 medications: buprenorphine, methadone, or naltrexone.^7^ These medications have demonstrated effectiveness, with associated reductions in opioid use and overdoses in the general population,^8^ as well as reduced overdose deaths and increased use of community-based treatment among recently detained populations.^9,10^ However, studies conducted in 2019 to 2020 found that less than a third of jails nationwide made MOUD available to all individuals with an opioid use disorder,^11,12,13^ with common barriers being cost, insurance, and other regulations.^11,13^

Recent federal guidance and policy changes present opportunities to increase the availability of MOUD in jails. Under the 2018 Substance Use Disorder Prevention That Promotes Opioid Recovery and Treatment for Patients and Communities (SUPPORT) Act, the Office of National Drug Control Policy was required to set drug-control policy priorities, including a National Drug Control Strategy and priorities for improving access to evidence-based treatment for individuals in the criminal justice system.^14^ Federal guidance based on the SUPPORT Act and the Americans With Disabilities Act further underscores MOUD as a priority for addressing the opioid crisis by protecting individuals taking MOUD from discrimination.^15^ Similarly, guidance from the American Society of Addiction Medicine^16^ and US Department of Justice and National Institute of Corrections^17^ calls for MOUD to be made available to all individuals with an OUD while in jail. This guidance also emphasizes the importance of coordinating treatment after release, a period during which individuals are at high risk of overdose. Recent 1115(b) Medicaid waivers emphasize improved services for persons living with OUDs in carceral settings and improved linkage to services after release.^18^ Implementation of this guidance is likely to vary by states and localities and over time, making measuring current MOUD provision in local jails important for understanding changes in these practices.

This study used a nationally representative survey to assess whether and for whom treatment for OUD was available in US jail settings. We also analyzed the association of characteristics of jails and communities in which they were located with the availability of MOUD within jails.

## Methods

Between June 6, 2022, and April 30, 2023, we conducted a cross-sectional, nationally representative survey study of local jails on their provision of SUD treatment services. This study received a determination as not human participants research from the Institutional Review Board at NORC at the University of Chicago and so was exempted from review. Surveys sent to jails included informed consent language. The study followed the American Association for Public Opinion Research (AAPOR) reporting guideline for surveys.

### Sample

A random sample of 2791 jails stratified by US Census region was invited to participate. The sample frame was created by combining and deduplicating the National Institute of Corrections National Compendium of Jails^19^ and Bureau of Justice Statistics list from the Census of Jails.^20^ Any jails or detention facilities that held individuals prior to or after sentencing were eligible; prisons were not eligible. The sample was selected to be representative of more than 3500 jails in the US, and the participating sample was weighted to adjust our data to the distribution of jails in each Census region and for jail-level nonresponse to the survey.

### Data Collection

The survey was administered via mail, the internet (email and QR code), and phone. All selected jails received an invitation postcard and email (if available) with the online survey link. Invitation materials noted the funding source and that staff knowledgeable about SUD screening and treatment should take the survey, multiple staff may be needed, and national jail and justice associations endorsed the survey. Nonresponding jails received an additional postcard, multiple email reminders, and up to 3 paper surveys in the mail.

### Measures

The 25-minute survey consisted of 23 multiple choice questions developed based on the literature and input from leaders in the field to assess jail characteristics and procedures for screening and treatment for SUD, including MOUD (ie, buprenorphine, methadone, and naltrexone). Jails indicated the most recent 12-month period during which they could accurately provide information on their facility’s substance use services.

### Survey Data

#### Availability of Treatment

Participants were asked, “Is any kind of substance use treatment or recovery support available to people while they are in this jail?” to which they could respond *yes* or *no.* If responding yes, participants were asked to specify which type of treatment (eg, outpatient substance use treatment, therapeutic community within the correctional system, or self-help meetings) was available.

#### Availability of MOUD

Participants indicating that any type of treatment was available were asked, “Has medication assisted treatment (MAT) been available to individuals in this jail to treat their OUD in the past year?” (Although *MOUD* is preferred by most clinicians and researchers, *MAT* is the acronym most familiar to jail staff and was therefore used in the survey.) The survey defined MAT as “the use of medications, often in combination with behavioral therapies, to provide a whole-patient approach to the treatment of opioid use disorder. Medications used include Buprenorphine, Methadone, and Naltrexone.” Respondents could indicate *yes*, *no*, or *don’t know*. For this analysis, jails that indicated *don’t know* were grouped with those that did not offer MOUD. If jails indicated that MOUD was available, they were also asked to report which type of medication and whether it was available to anyone with an OUD or only to specific groups (eg, pregnant people or individuals being released).

#### Jail Size

Jails were asked to report their mean daily population. They were instructed to provide an estimate if they did not have an exact count available.

#### Health Care Service Model

Because the ability to offer MOUD depends in part on health care staff, respondents were asked to indicate their health care delivery model. Available categories were direct services (all health care services provided by jail employees), contracted (all health care services provided by contracted vendors or clinicians), hybrid (a combination of direct and contracted), or other.

### County-Level Data

To understand the communities in which jails were located, we used publicly available county-level data from the Opioid Environment Policy Scan (OEPS) database.^21^ We measured opioid overdoses, treatment availability, urbanicity, and socioeconomic factors.

#### Overdose Mortality Rate

The overdose mortality rate variable included deaths from an opioid overdose per 100 000 persons for the year 2020. Data were sourced from the Centers for Disease Control and Prevention (CDC) National Center for Health Statistics via the OEPS database.

#### Access to Treatment

Because access to MOUD in the community is critical for people after release from jail and jails may partner with outside vendors or clinicians,^22^ we measured access to treatment by using mean drive time to the nearest facility providing MOUD. The OEPS database calculates the mean drive time from the center of each census tract to facilities providing buprenorphine, methadone, and naltrexone identified using the Substance Abuse and Mental Health Services Administration (SAMHSA) treatment locator.^23^ We used the lowest mean drive time to the closest facility providing MOUD in our analyses.

#### Urbanicity

The National Center for Health Statistics rural-urban classification scheme^24^ defines 6 categories of counties: (1) large central metropolitan counties with a population of 1 million residents or more and 250 000 residents within the principal city, (2) large fringe metropolitan counties with a population of 1 million residents or more that do not qualify as central, (3) medium metropolitan counties with a population of 250 000 to 999 999 residents, (4) small metropolitan counties with a population of less than 250 000 residents, (5) micropolitan counties with an urban cluster of 10 000 or more residents, and (6) noncore counties, or counties that do not have an urban cluster of at least 10 000 residents. We used 5 categories in our analysis; large central and large fringe categories were combined into 1 large metropolitan category.

#### County Socioeconomic Factors

To assess socioeconomic characteristics of the counties in which jails were located, we used the CDC 2020 Social Vulnerability Index summary ranking, which ranks counties based on 16 social factors, such as poverty level, unemployment, education, housing and transportation. It is provided as a percentile that compares a county with other counties. A lower percentile indicates less vulnerability.^25^

#### Region

Jails were classified into 1 of the 4 US Census regions based on zip code. These categories were Northeast, Midwest, South, and West.

### Statistical Analysis

Analyses were conducted using SPSS statistical software version 29.0 (IBM); 95% CIs for proportions were computed using SAS Enterprise Guide statistical software version 8.3.8 (SAS Institute). Only jails with complete data for all variables of interest were included in analyses. Descriptive weighted statistics were computed for characteristics of jails and availability of treatment and MOUD. We conducted 3 separate binary logistic regressions with statistical weights: availability of any SUD treatment, availability of any MOUD, and availability of MOUD for anyone with an OUD. Variables were selected for inclusion in the multivariable binary logistic regression based on a priori hypotheses and associations at the bivariate level using a 2-sided *P* < .05 threshold.

## Results

### Description of Participating Jails (Weighted)

Among 2791 invited jails, a total of 1028 unique jails completed the survey, for a response rate of 36.8%, which is comparable to similar studies with jails.^13,26^ After merging the sample with county data, 927 jails were included in the analysis, representative of 3157 jails nationally after weighting. As shown in Table 1, most jails were located in nonmetropolitan areas (ie, micropolitan or noncore areas; 1756 jails [55.6%; 95% CI, 52.3% to 59.0%]) and offered contracted health care services (1886 jails [59.7%; 95% CI, 56.5% to 63.0%]). Among 278 jails that indicated another noncontracted or non–direct-service arrangement (8.8%; 95% CI, 6.9% to 10.7%), most jails specified that they transported people to a health care facility as needed. Some jails housed males only (230 jails [7.3%; 95% CI, 5.5% to 9.1%]), and a few held females only (7 jails [0.2%; 95% CI, <0.1% to 0.5%]), possibly aligning with the 14% female jail population.^27^

**Table 1.** Characteristics of Participating US Jails, June 2022 to April 2023

Characteristic	Jails, No. (% [95% CI]) (N = 3157)^a^
Jail population	
Male only	230 (7.3 [5.5 to 9.1])
Female only	7 (0.2 [<0.1 to 0.5])
Male and female	2919 (92.5 [90.6 to 94.3)
Region	
Northeast	217 (6.9 [5.4 to 8.3])
Midwest	1021 (32.3 [29.4 to 35.3])
South	1444 (45.7 [42.3 to 49.2])
West	475 (15.1 [13.0 to 17.1])
Urbanicity^b^	
Large metropolitan	528 (16.7 [14.2 to 19.2)
Medium metropolitan	469 (14.9 [12.4 to 17.3])
Small metropolitan	404 (12.8 [10.5 to 15.0])
Micropolitan	686 (21.7 [19.0 to 24.5])
Noncore	1070 (33.9 [30.7 to 37.1])
Health care model	
Direct services	317 (10.1 [8.0 to 12.0])
Contracted services	1886 (59.7 [56.5 to 63.0])
Hybrid (combination of direct and contracted)	675 (21.4 [18.7 to 24.1])
Other noncontracted or nondirect arrangement	278 (8.8 [6.9 to 10.7])
Mean daily population	
0-25	752 (23.8 [21.0 to 26.6])
26-50	385 (12.2 [10.0 to 14.4])
51-100	560 (17.7 [15.2 to 20.3])
101-200	564 (17.9 [15.3 to 20.4])
≥200	896 (28.4 [25.3 to 31.5])
County characteristics, mean (SD)	
Opioid overdose mortality rate (2020), per 100 000	24.8 (13.5)
Social Vulnerability Index summary ranking	0.51 (0.28)
Mean driving time to nearest MOUD, min	19.3 (18.5)

Abbreviation: MOUD, medications for opioid use disorder.

^a^ Data are weighted. The analysis of 927 jails was representative of 3157 jails nationally after weighting.

^b^ The breakdown is based on the National Center for Health Statistics classification scheme.^24^

For all 2791 invited jails, we compared available information on nonrespondents with that of respondents and found no difference based on urbanicity (χ^2^ = 2.5; *P* = .29). Participating jails had a lower mean (SD) number of adult males admitted compared with nonrespondents (172.3 [324.3] vs 214.2 [412.4] males; *t*_2396_ = 2.6; *P* = .004). We also found regional variations, with fewer jails in the South (342 of 1129 unweighted jails [30.3%; 95% CI, 27.6% to 33.0%]) completing the survey than in other regions (Midwest: 377 of 870 unweighted jails [43.3%; 95% CI, 40.0% to 46.6%]; Northeast: 98 of 253 unweighted jails [38.7%; 95% CI, 32.7% to 44.8%]; West: 211 of 539 unweighted jails [39.2%, 95% CI, 35.0% to 43.3%]; χ^2^ = 38.2; *P* < .001).

### Availability of Treatment for SUDs

Among 3157 jails represented after weighting, less than half of jails (1383 jails [43.8%; 95% CI, 40.5%-47.1%]) offered some type of MOUD, while 405 jails (12.8%; 95% CI, 40.5%-47.1%) offered at least 1 medication to anyone with an OUD. Among 830 jails (37.5%; 95% CI, 33.6%-41.4%) not offering MOUD, the most common reason indicated was lack of adequate licensed staff (413 jails [49.8%; 95% CI, 43.1%-56.6%]). Most jails offered some type of SUD treatment or recovery support (2213 jails [70.1%; 95% CI, 66.9%-73.2%]). Table 2 summarizes the availability of treatment and reasons for not offering MOUD.

**Table 2.** Availability of Treatment for SUDs in US Jails, June 2022 to April 2023

Type of treatment available	Jails, No. (% [95% CI]) (N = 3157)^a^
Any type of SUD treatment or recovery support	2213 (70.1 [66.9-73.2])
Self-help meetings (eg, Alcoholics Anonymous or SMART Recovery)	1388 (62.7[58.8-66.5])^b^
Services for co-occurring substance use and mental health conditions by a licensed clinician	1070 (48.4[44.4-52.3])^b^
Therapeutic community within the correctional system by a licensed clinician	763 (34.5 [30.7-38.2])^b^
Outpatient SUD treatment by a licensed clinician	729 (33.0 [29.3-36.6])^b^
Other treatment or recovery services	612 (27.7[24.1-31.3])^b^
MOUD	1383 (43.8 [40.5-47.1])
Buprenorphine	966 (69.9 [65.3-74.6])^c^
For anyone with an OUD who requests it	265 (27.5 [22.4-32.5])^d^
For pregnant individuals	387 (40.1 [34.4-45.7])^d^
For people already receiving buprenorphine when booked	691 (71.5 [66.2-76.8])^d^
For individuals being released	225 (23.3 [18.6-27.9])^d^
For other persons	211 (21.8 [17.2-26.4])^d^
Methadone	644 (46.6 [41.7-51.5])^c^
For anyone with an OUD who requests it	74 (11.6 [7.0-16.1])^e^
For pregnant individuals	208 (32.2 [25.4-39.0])^e^
For people already receiving methadone when booked	506 (78.5 [72.4-84.6])^e^
For individuals being released	49 (7.6 [4.0-11.2])^e^
For other persons	100 (15.5 [10.5-20.5])^e^
Naltrexone	753 (54.5 [49.5-59.4])^c^
For anyone with an OUD who requests it	277 (36.8 [30.4-43.0])^f^
For people already receiving naltrexone when booked	390 (51.7 [45.3-58.2])^f^
For individuals being released	366 (48.6 [42.2-55.1])^f^
For other persons	157 (20.8 [15.5-26.2])^f^
≥1 Type of MOUD available to anyone with an OUD who requests it	405 (12.8 [10.7-14.9])
MOUD is not available (other type of treatment is available)	830 (37.5 [33.6-41.4])^b^
Because jail does not have adequate staffing or staffing licensed to provide MOUD	413 (49.8 [43.1-56.6])^g^
Because policies prevent jail from offering MOUD	152 (18.3 [12.9-23.7])^g^
Because MOUD is too expensive or budget does not allow	132 (15.9 [10.9-20.8])^g^
Because jail does not see many individuals with OUD	103 (12.4 [8.0-16.8])^g^
Other reasons for not offering MOUD	198 (23.9 [18.2-29.6])^g^

Abbreviations: MOUD, medications for opioid use disorder; SMART, Self-Management and Recovery Training; SUD, substance use disorder.

^a^ Data are weighted, and weighted numbers are reported. The analysis of 927 jails was representative of 3157 jails nationally after weighting.

^b^ The percentage is among jails that indicated that they offered some type of treatment for SUDs.

^c^ The percentage is among jails that indicated that MOUD was available.

^d^ The percentage is among jails that indicated that buprenorphine was available.

^e^ The percentage is among jails that indicated that methadone was available.

^f^ The percentage is among jails that indicated that naltrexone was available.

^g^ The percentage is among jails that did not offer MOUD but did offer some type of treatment.

As shown in Table 3, regression analyses found that offering any SUD treatment, and MOUD specifically, differed based on the jail health care services model, size, and region, as well as social vulnerability and MOUD availability within the surrounding county. While urbanicity and county opioid overdose mortality rate were not associated with the availability of MOUD in jails, county opioid overdose mortality rate was associated with a greater likelihood of offering any type of SUD treatment (adjusted odds ratio [aOR] per 1-unit [death/10 000 population] increase, 1.21; 95% CI, 1.10-1.34).

**Table 3.** Factors Associated With Availability of MOUD in 3157 US Jails, June 2022 to April 2023^a^

Independent variable	Availability of any treatment, aOR (95% CI)	*P* value	Availability of MOUD, aOR (95% CI)	*P* value	Availability of MOUD to anyone who requests it, aOR (95% CI)	*P* value
Urbanization						
Noncore	[1 Reference]	NA	[1 Reference]	NA	[1 Reference]	NA
Large metropolitan	0.54 (0.40-0.72)	<.001	0.86 (0.65-1.14)	.30	1.49 (0.98-2.26)	.06
Medium metropolitan	1.70 (1.23-2.35)	.001	1.31 (1.00-1.73)	.05	1.43 (0.94-2.17)	.10
Small metropolitan	0.98 (0.72-1.34)	.90	0.76 (0.57-1.02)	.07	0.73 (0.46-1.16)	.18
Micropolitan	0.92 (0.72-1.17)	.48	1.10 (0.87-1.38)	.42	1.07 (0.73-1.57)	.74
Health care model						
Direct services	1 [Reference]	NA	[1 Reference]	NA	[1 Reference]	NA
Contracted services	1.14 (0.85-1.52)	.38	1.06 (0.81-1.39)	.67	0.71 (0.49-1.03)	.07
Hybrid (combination of direct and contracted)	1.31 (0.94-1.82)	.11	1.12 (0.83-1.51)	.47	0.70 (0.46-1.07)	.10
Other noncontracted or nondirect arrangement	0.51 (0.34-0.76)	.001	0.39 (0.25-0.61)	<.001	0.17 (0.07-0.46)	<.001
Mean daily jail population						
0-25	1 [Reference]	NA	[1 Reference]	NA	[1 Reference]	NA
26-50	2.46 (1.83-3.32)	<.001	1.56 (1.16-2.10)	.003	1.39 (0.84-2.32)	.20
51-100	2.80 (2.12-3.71)	<.001	1.76 (1.33-2.31)	<.001	1.95 (1.22-3.11)	.005
101-200	2.89 (2.16-3.85)	<.001	2.21 (1.67-2.93)	<.001	2.11 (1.32-3.38)	.002
≥200	6.72 (5.00-9.04)	<.001	4.97 (3.75-6.58)	<.001	3.05 (1.93-4.84)	<.001
Region						
South	1 [Reference]	NA	[1 Reference]	NA	[1 Reference]	NA
Midwest	2.18 (1.72-2.77)	<.001	2.61 (2.10-3.25)	<.001	1.92 (1.37-2.69)	<.001
Northeast	20.05 (8.17-49.16)	<.001	9.79 (6.55-14.61)	<.001	15.70 (10.73-22.98)	<.001
West	2.63(1.99-3.46)	<.001	3.23 (2.53-4.11)	<.001	4.41 (3.15-6.17)	<.001
2020 County opioid overdose mortality rate, per 1-SD increase^b^	1.21 (1.10-1.34)	<.001	1.04 (.96-1.14)	.33	1.03 (0.91-1.18)	.60
Social Vulnerability Index summary rank, per 1-percentile increase	0.28 (0.19-0.40)	<.001	0.45 (0.32-0.64)	<.001	0.35 (0.21-0.59)	<.001
Mean drive time to closest facility providing MOUD in county, minutes, per 1-SD increase^b^	0.80 (0.73-0.88)	<.001	0.72 (0.64-0.80)	<.001	0.69 (0.55-0.85)	.001

Abbreviations: aOR, adjusted odds ratio; MOUD, medications for opioid use disorder; NA, not applicable.

^a^ Results are presented from 3 logistic regressions assessing the availability of any type of treatment, availability of MOUD, and availability of MOUD for anyone who requested it using weighted data. The analysis of 927 jails was representative of 3157 jails nationally after weighting.

^b^ *Z* scores were computed for opioid overdose mortality rate and mean drive time to the closest facility providing MOUD in the county to more clearly show outcomes for these continuous variables with large SDs.

We found no significant difference in the likelihood of offering these treatments between jails with direct, contracted, or hybrid service arrangements. However, compared with jails with direct health care services, jails with other (ie, neither contracted nor direct) health care service arrangements displayed lower odds of offering treatment and medications (aOR, 0.51; 95% CI, 0.34-0.76 for any SUD treatment; aOR, 0.39; 95% CI, 0.25-0.61 for any MOUD; aOR, 0.17; 95% CI, 0.07-0.46 for MOUD for any individual).

The likelihood of offering SUD treatment and MOUD increased with jail size and decreased with higher community social vulnerability. Each 1-percentile increase in social vulnerability was associated with a decrease in the odds of offering any treatment (aOR, 0.28; 95% CI, 0.19-0.40), with accompanying decreases in the odds of offering any MOUD (aOR, 0.45; 95% CI, 0.32-0.64) or offering MOUD to anyone with an OUD (aOR. 0.35; 95% CI, 0.21-0.59). Per every 1 SD (18 minutes) of mean drive time above the mean (19.3 minutes) to a facility providing MOUD in the county, there was an associated decrease in the odds of the jail offering any type of SUD treatment (aOR, 0.80; 95% CI, 0.72-0.88), any MOUD (aOR, 0.72; 95% CI, 0.64-0.80), and any MOUD to anyone with an OUD (aOR, 0.69; 95% CI, 0.55-0.85).

## Discussion

Providing evidence-based medications for the treatment of OUD in correctional settings is associated with improved outcomes and reduced opioid-related deaths.^9,10,28^ However, this survey study found that less than half of jails nationwide had MOUD available within their facilities (43.8%) and very few (12.8%) offered it to anyone with an OUD. Among jails with MOUD available, the most common type of medication used was buprenorphine (69.9%), followed by naltrexone (54.5%), while less than half of jails offered methadone (46.6%). Most jails offered some type of substance use treatment or recovery support (70.1%).

While variations in methods and survey questions do not allow for a straightforward comparison of our results with those of other jail surveys conducted in 2019 to 2021, our data are consistent with earlier findings.^11,12,13^ Even when MOUD is available in jails, it is not universally available to anyone with an OUD as current guidance recommends.^16,17^ With the exception of pregnant persons and individuals already receiving MOUD, most detainees with OUD are unlikely to have access to MOUD.^11^

Given the association between opioid use and involvement with the legal system,^4,5,6^ these findings highlight a missed opportunity for reducing the impact of the opioid crisis on communities. Our data offer insight into several factors contributing to this gap in care that together suggest that resource challenges in jails and the communities in which they are located may be preventing individuals in most need from accessing this evidence-based treatment.

The most common reason jails reported for not offering MOUD was a lack of adequate or licensed staff to administer it. In support of this finding, we found that the type of health care model was associated with offering MOUD or any type of treatment for SUDs, and jails reporting services other than direct, contracted, or hybrid health care arrangements (generally, jails with no on-site health care services available) were less likely to offer MOUD than those using their own health care staff. MOUD services require ready access to licensed health care clinicians; these staff present added cost and logistical barriers for many jails. Contracted and hybrid options may offer different cost structures while maintaining MOUD availability, and the National Drug Control Strategy goal to reduce the shortage of behavioral health clinicians may further assist jails with their staffing challenges.^14^

We did not find an association between county opioid overdose mortality rate and the availability of MOUD in the jail. Other county-level factors highlight the importance of community context. The availability of MOUD in the surrounding community was associated with their use in jails. Jails located in counties with fewer accessible facilities providing MOUD (ie, longer mean driving times to facilities with MOUD) were less likely to have MOUD available, suggesting that these jails may face challenges with finding treatment partners or ensuring continuity of treatment after release.

Although logistically challenging, partnering with local facilities providing MOUD may help jails make treatment available for their detainees.^22,29^ Similarly, larger jails were more likely to offer SUD treatment than smaller jails, potentially owing to their location in more populated areas with more resources. As a measure that integrates factors like poverty level, unemployment, education, and racial and ethnic minority status, higher social vulnerability levels being associated with lower availability of MOUD in jails emphasizes the connection with contextual factors in the community. The finding is also consistent with previous research,^30^ including recent analyses underscoring the dependence of criminal-legal system actors on the broader policy environment. For example, a 2023 analysis^31^ of MOUD provision through problem-solving courts found higher provision in states that expanded Medicaid under the Affordable Care Act. Such results are consistent with our finding that jails located in southern states had lower MOUD availability.

Together, these findings suggest that jails’ local community context is associated with the availability of MOUD to the jail population. Because the external environment is associated with the ability to receive MOUD within the jail and after release, a highly vulnerable time for someone recovering from an OUD, our findings underscore that efforts to improve access to treatment are dependent on shared resources and relationships across public safety and public health contexts.

### Limitations

Our study has several limitations. First, while comparable with other surveys on MOUD in jails, our study had a modest response rate of 36.8%. Thus, the availability of MOUD in jails may differ from that reported in our study. Reasons for nonresponse could have been time limitations or reluctance to report unavailability of MOUD. We found no difference between nonrespondents and respondents based on urbanicity and only small differences in jail size and region. Second, like in any self-report survey, underreporting of undesirable results (eg, the absence of MOUD) was possible. We attempted to minimize the impact of this issue by promising confidentiality. Third, we used mean drive times to assess MOUD accessibility, which do not fully address access constraints and depend on the accuracy of the SAMHSA treatment locator; however, this approach is consistent with prior MOUD access research.^32,33^ Fourth, our study represents 1 cross section in time. Future research should monitor change over time with the evolving nature of the opioid crisis and related policies.

## Conclusions

Correctional systems are intimately connected with the communities in which they reside. The vast majority of individuals in jail will return to these communities, and those who have not received effective SUD treatment while detained will return at greatly heightened risk for overdose in the weeks immediately after their release. In contrast, a recent modeling analysis estimated that postincarceration overdose deaths could be reduced as much as 31% if jails made all 3 forms of MOUD available to all detainees with OUD.^34^ Jails are thus positioned to play key roles in curbing the opioid crisis. In this national survey study, relatively few jails indicated offering MOUD, the frontline treatment for OUD. Increasing resources for health care services in jails and expanding MOUD availability in communities are likely necessary first steps given that our data highlight the importance of the community context surrounding the jail. While some policies have been implemented to expand access,^35^ policies that improve clinician reimbursement and expand Medicaid coverage for MOUD are essential to support frontline treatment for incarcerated persons and others at risk in the overdose epidemic.

## Notes

Supplement.
Data Sharing Statement
