# Supplementary material for: Factors Associated With the Availability of Medications for Opioid Use Disorder in US Jails
Source: JAMA Netw Open. 2024 Sep 24;7(9):e2434704. doi: 10.1001/jamanetworkopen.2024.34704 (PMC11423166; doi:10.1001/jamanetworkopen.2024.34704)
Supplement: Supplement. — Data Sharing Statement [file jamanetwopen-e2434704-s001.pdf]

Data Sharing Statement

Flanagan Balawajder. Factors Associated With the Availability of Medications for Opioid Use Disorder in US Jails. *JAMA Netw Open*. Published online September 24, 2024. doi:10.1001/jamanetworkopen.2024.34704

Data
Data available: Yes
Data types: Deidentified participant data
How to access data: Deidentified participant data will be made available upon request
When available: beginning date: 09-24-2024

Supporting Documents
Document types: None

Additional Information
Who can access the data: It will be made publicly available upon request. Date above is estimated.
Types of analyses: Deidentified data will be made publicly available upon request.
Mechanisms of data availability: Deidentified data will be made available upon request.
